# Implications of Antiphospholipid and Antineutrophilic Cytoplasmic Antibodies in the Context of Postinfectious Glomerulonephritis

**DOI:** 10.1155/2017/9896210

**Published:** 2017-02-01

**Authors:** Daniel Leifer, Lavjay Butani

**Affiliations:** ^1^Department of Pediatrics, Northwestern University Feinberg School of Medicine, Chicago, IL, USA; ^2^Department of Pediatrics, University of California Davis School of Medicine, Sacramento, CA, USA

## Abstract

While antineutrophil cytoplasmic antibody (ANCA) positivity has been documented in some patients with postinfectious glomerulonephritis (PIGN) and is associated with more severe disease, antiphospholipid antibodies (APA) are not known to be a common occurrence. We describe a child with severe acute kidney injury who was noted to have prolonged positivity of both ANCA and APA; a renal biopsy showed noncrescentic immune complex mediated glomerulonephritis with subepithelial deposits compatible with PIGN. He recovered without maintenance immunosuppressive therapy and at last follow-up had normal renal function. We discuss the cooccurrence and implications of ANCA and APA in children with PIGN.

## 1. Background

Approximately 90% of patients with active granulomatosis with polyangiitis, 70% of patients with microscopic polyangiitis, and 50% with Churg-Strauss syndrome are ANCA positive [1]. In addition to pauci-immune vasculitis, ANCA positivity has also been described in other glomerular diseases including lupus nephritis, antiglomerular basement membrane antibody disease, and much less commonly postinfectious glomerulonephritis (PIGN) [2]. Anticardiolipin (aCL) antibodies have been described predominantly in the setting of lupus and primary antiphospholipid antibody syndrome and are associated with a thrombotic risk; a single report documented a high incidence of aCL antibodies during the acute and convalescence periods of poststreptococcal glomerulonephritis (PSGN), especially in children [3]. There are no published reports on the lupus anticoagulant or APA in the setting of PIGN. We report our experience with a child with severe PIGN who had prolonged ANCA and APA positivity and who recovered renal function with minimal immunosuppression.

## 2. Case Report

An 8-year-old boy presented with severe oliguric acute kidney injury after 10 days of vomiting. Eight days prior to admission he was started on amoxicillin for acute otitis media and sinusitis. The day prior to admission, due to persistent vomiting, laboratory testing was obtained and revealed heavy proteinuria and microscopic hematuria; serum chemistries showed a potassium level of 6.3 mmol/L (6.3 mEq/L), a blood urea nitrogen level of 58.5 mmol/L (164 mg/dL), and a serum creatinine level of 884 *μ*mmol/L (10 mg/dL). There was no history of fever, sore throat, skin rash, or arthritis. Examination was notable for blood pressure of 143/81 mm Hg, bulging of the right tympanic membrane, and mild edema of both legs. Serologic testing revealed negative Hepatitis serologies; his antinuclear antibody (ANA) was positive at low titer of 1 : 80 and his ANCA IgG was positive at 1 : 160 with a peripheral ANCA (p-ANCA) staining pattern. Based on these preliminary results, the patient was started on hemodialysis and plasmapheresis and received high-dose intravenous methylprednisolone. Further testing revealed that his antimyeloperoxidase antibodies (MPO) and serine protease 3 (PR3) antibodies were both negative, as was his antidouble stranded DNA antibody. An APA assay showed an elevated IgM level for *β*-2-glycoprotein I antibody (64.1 MPL, normal < 15); aCL antibodies were not detected and a Dilute Russel Viper Venom Time (DRVVT) was normal. The complement C4 level was normal but his C3 level was low at 0.07 g/L (7 mg/dL) (normal 70–206 mg/dL). The antistreptolysin O titer and the streptozyme screen were both negative, as was the anti-DNAse B. Renal biopsy subsequently showed diffuse proliferative noncrescentic glomerulonephritis with “full house” immunofluorescence and numerous subepithelial hump-like deposits with scattered subendothelial and mesangial deposits. Based on a diagnosis of severe PIGN and since his urine output has improved, his plasmapheresis was discontinued after 3 treatments and his steroids rapidly tapered. He was discharged after 11 days with improving real function.

The complement C3 and serum creatinine normalized by 2 weeks; his urinalysis remained abnormal for much longer and had normalized by 11 months. Other serologic testing took longer to normalize. His ANCA took 4 months to normalize; his ANA remained mildly elevated (1 : 40 to 1 : 80) until 2 years after presentation; the last test to normalize was the *β*-2-glycoprotein I antibody IgM that had remained quite high (39.6–97.7 MPL, normal < 15) and only became negative 3.5 years later. Serial aCL antibody tests remained negative. His DRVVT, which was normal at admission, was elevated at 11 months and 23 months, consistent with the presence of lupus anticoagulant; this normalized 3.5 years after admission. At the patient's last visit, 3.5 years after admission he was in good health and not on any medications.

## 3. Discussion

p-ANCA positivity is a rare and poorly understood occurrence in PIGN. Ardiles et al. studied 210 patients with PSGN and found 18 (9%) to be ANCA positive [2]. Of these, only 4 (all children) were p-ANCA positive while the rest had an atypical staining pattern. None of the p-ANCA positive patients had autoantibodies against PR3 and only 1 had an antibody against MPO. In their study, ANCA positivity correlated with a more severe disease course of PSGN. Kanai et al. described a pediatric patient with severe crescentic PSGN who also had p-ANCA positivity and MPO positivity [4]; at 18 months, the patient's renal function had improved on long-term azathioprine and oral prednisolone, although MPO titers remained elevated. They and others postulated that MPO positivity may correlate with crescent formation [5]. APA levels were not noted in either report.

Our patient had a prolonged elevation in ANCA and *β*-2-glycoprotein I antibody and low titer ANA, as well as intermittent evidence of the lupus anticoagulant, which have never been reported before in the setting of PIGN. It is not clear whether this generalized autoimmunity was precipitated by his renal injury or was the result of the infection that triggered the glomerulonephritis. ANCA have been postulated to be triggered by infectious agents, especially* Staphylococcus aureus* which has several PR3 encoding gene complementary sequences [1]. The presence of multiple antibodies could also indicate polyclonal B cell stimulation due to molecular mimicry or unchecked neutrophil apoptosis, resulting from the triggering infection. Lastly these may have been incidental abnormalities, since a very small proportion of healthy people, when screened, will have ANCA or APA; however, this is typically seen in adults and, moreover, would not be expected to resolve in parallel with an improvement in our patient's clinical and laboratory test abnormalities. APA and also antibodies against *β*-2-glycoprotein I have been noted in patients with viral, bacterial, and parasitic infections and are often transient [6]; these antibodies have been hypothesized to have a role in innate immunity, but they can be associated with a thrombotic risk if persistently elevated, are proinflammatory, and have the potential to exacerbate inflammation-mediated tissue injury [7].

Although our patient was started on plasmapheresis and corticosteroids while biopsy results were awaited; the plasmapheresis was stopped abruptly and steroids were quickly tapered down. Steroids have no role in uncomplicated PIGN and even in severe crescentic PIGN, their role is controversial.

Through this case we would like to bring to the attention of pediatric nephrologists to the possible cooccurrence of ANCA and APA in the setting of PIGN, especially children presenting with severe disease. Whether these have a pathogenic role in increasing the severity of tissue injury remains to be determined; however, if persistent and when associated with progressively worsening disease, consideration should be given to measuring them and possibly using immunosuppression and/or plasmapheresis to help in renal recovery.
